# An implantable compound-releasing capsule triggered on demand by ultrasound

**DOI:** 10.1038/srep22803

**Published:** 2016-03-11

**Authors:** Olga Ordeig, Sau Yin Chin, Sohyun Kim, Parag V. Chitnis, Samuel K. Sia

**Affiliations:** 1 Department of Biomedical Engineering, Columbia University, 351 Engineering Terrace, 1210 Amsterdam Avenue, New York, NY 10027, United States; 2 Department of Bioengineering, George Mason University, 4400 University Drive, Fairfax, VA 22032, United States; 3 F. L. Lizzi Center for Biomedical Engineering, Riverside Research, New York, NY 10038, United States

## Abstract

Implantable devices have a large potential to improve human health, but they are often made of biofouling materials that necessitate special coatings, rely on electrical connections for external communication, and require a continuous power source. This paper demonstrates an alternative platform, which we call iTAG (implantable thermally actuated gel), where an implanted capsule can be wirelessly controlled by ultrasound to trigger the release of compounds. We constructed a millimeter-sized capsule containing a co-polymer gel (NiPAAm-co-AAm) that contracts above body temperature (i.e. at 45 °C) to release compounds through an opening. This gel-containing capsule is biocompatible and free of toxic electronic or battery components. An ultrasound hardware, with a focused ultrasound (FUS) transducer and a co-axial A-mode imaging transducer, was used to image the capsule (to monitor in real time its position, temperature, and effectiveness of dose delivery), as well as to trigger a rapid local rise in temperature, contraction of gel, and release of compounds *in vitro* and *in vivo*. The combination of this gel-based capsule and compact ultrasound hardware can serve as a platform for triggering local release of compounds, including potentially in deep tissue, to achieve tailored personalized therapy.

In an era of personalized medicine, it will be desirable to tailor a therapeutic dose in a manner that is rapidly responsive to the progression of disease^1^. The ability to alter the dosing amount and schedule in real time could be especially attractive when implemented in implantable devices^2^,^3^.

Towards this goal, current implantable devices^4^,^5^ exhibit several limitations: 1) they consist of biofouling materials that necessitate special coatings^6^; 2) they contain electronics or wires for external communication that can be cumbersome for the patient; and 3) they require a continuous power source such as a battery which can limit the lifespan and utility of the device^6^,^7^. Recent technologies that adapt CMOS-based electronics, including surface-skin sensors and implanted electronics^8^, require a power supply or electrical interconnections between the interior and exterior of the body. By comparison, while implantable technologies that rely on magnetic input or infrared light do not require electronic components, the precision of targeting and triggering the implantable device deteriorate with increase in depth. Another recent technology uses resorbable electronic circuits with wireless communication^9^ but requires continuous power.

Here, we aim to establish a proof of concept for an alternative platform, which uses ultrasound imaging, a commonly available and portable diagnostic imaging method, to trigger the release of compounds in implantable devices; and to demonstrate of its effective applicability *in vitro* and *in vivo*.

Ultrasound is an attractive modality for actuating components in microdevices for four reasons: 1) it is a well-established modality for noninvasively imaging deep anatomical sites (millimeters or even centimeters of dense tissue) with relatively fine resolution in real-time; 2) it uses nonionizing radiation and has an excellent safety record; 3) it can be made widely accessible to patients, even in outpatient clinics, due to its cost effectiveness and portability^10^; 4) it can focus energy into a small volume (less than 1 mm^3^), which can be used to achieve localized temperature changes^11^, while providing real-time temperature feedback^12^. Furthermore, technology development over the last decade has led to a rapid miniaturization of ultrasound instrumentation, such as a compact research ultrasound imaging system that is the size of a PC and can achieve acoustic outputs sufficient for thermal actuation of hydrogel-based implants is commercially available^13^. Such developments can facilitate remote operation by experts to make these techniques available to patients in rural or low-income areas.

Here, we employ focused ultrasound (FUS), which has been used for thermal ablation and more recently for localized drug delivery. Common methods for achieving drug delivery with FUS include thermal-based activation of drug-loaded liposomes^14^,^15^,^16^ and mechanical disruption of microbubbles carrying therapeutic payloads^17^,^18^,^19^. Some studies combined FUS with thermally-activated gels^20^,^21^, typically inside microscopic free-floating carriers. Here, we present an alternate strategy, called iTAG (implantable thermally actuated gel), of implanting devices at the disease site and using FUS to trigger local release. This strategy can potentially deliver a compound locally (rather than systematically), with the dosage being tunable in real time.

## Results

### Overall concept and fabrication of device

**Figure 1:**
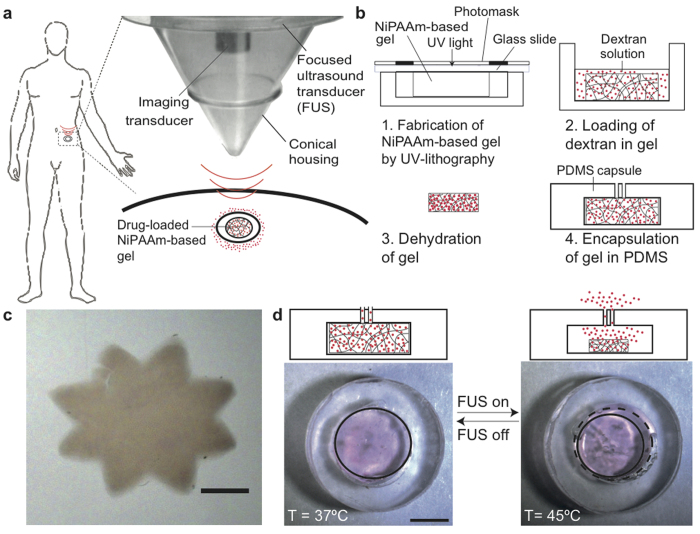
Ultrasound-based approach for triggering implanted devices. (**a**) Schematic diagram showing the use of focused ultrasound to trigger local temperature change and compound release, using thermally responsive components inside an implantable device. Picture of the FUS transducer used in this study. The man silhouette drawing belongs to SC. (**b**) Schematic diagram showing the fabrication steps for the implantable microdevice. (**c**) Picture of a NiPAAm-based gel (80:20 NiPAAm:AAM, 5% MBAAM) fabricated by UV lithography with dimensions on the micron range (scale bar: 500 μm). (**d**) Cross-section diagram (top) and top-down photograph (bottom) showing ~20% contraction of the gel across 8 °C (scale bar: 1 mm).

In our approach, FUS imparts energy into thermally responsive components inside an *in vivo*-implanted capsule (Fig. 1a). The capsule contains a thermally responsive hydrogel (NiPAAm-co-AAm) that can be imaged by ultrasound, and non-invasively squeezed or swollen in real time. This strategy integrates new methodologies in the delivery vehicle (i.e. fabrication of a gel-based capsule), and instrumentation which can image the capsule, trigger compound release, and control the set temperature with real-time feedback. NiPAAm-co-AAM co-polymer was chosen based on its thermal properties as well as its biocompatibility. A previous study studied the possible toxic effects of injecting NiPAAM-co-AAM in its aqueous form to pregnant mice and concluded that this oligomer is not shown to possess significant toxic effects^22^.

We used UV photo-polymerization to fabricate a 6.5-mm diameter disc made of N-isopropylacrylamide (NiPAAm)-co-acrylamide (AAm) (Fig. 1b). Briefly, we dissolved a pre-polymer solution mixture containing NiPAAm, co-monomer AAm, crosslinker MBAAm, and the photoinitiator Daracour 1173 in water:ethanol (50:50 v/v), and placed the mixture inside a PDMS well and covered by a glass slide. A photomask with the desired design (e.g. with different diameters of the gels) was placed over the glass slide, and the photo-polymerization was initiated by irradiating with UV light. We rinsed the hydrogels to remove the non-crosslinked pre-polymers. By changing the designs in the photomasks, we successfully fabricated NiPAAm-based gels with features less than 500 μm (Fig. 1c), consistent with the requirements of many implanted microdevices^23^ (if desired, we expect further optimization in gel composition and exposure conditions could significantly decrease feature sizes). To track release of compounds, we loaded the NiPAAm:AAm copolymers with a fluorescent labeled dextran by drying the gels, re-swelling in a concentrated solution of dextran, and re-drying (Fig. 1d; see Supporting Information Online for details). This dextran-loaded NiPAAm:AAm gel was then encapsulated in a PDMS capsule with two small openings (160 μm in diameter for this design).

### Characterization of gel contraction and compound release

**Figure 2:**
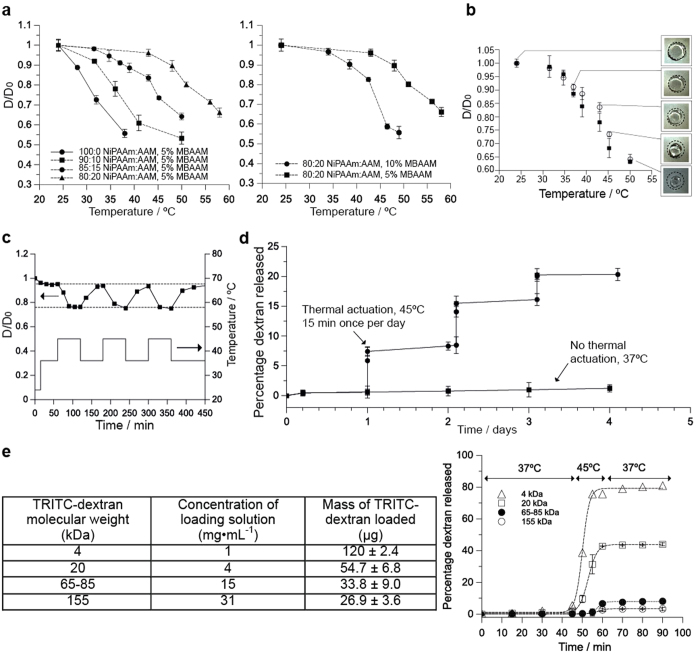
Temperature-triggered changes in gel behavior. (**a**) Relative decrease of the NiPAAm:AAm gel diameter as a function of different NiPAAm:AAM ratios (left), and as a function of different MBAAM percentages (right). Experiments were performed in water. (**b**) Relative decrease of the gel diameter (for 85:15 w/v NiPAAm:AAm, 5% MBAAM) in water (○) and in PBS buffer (■) when the temperature is changed from room temperature to 50 °C using a water bath. The diameter of the gel at 24 °C (Do) was 7.3 ± 0.08 mm in water and 7.2 ± 0.11 mm in PBS. All data points correspond to the average of at least 3 different experiments and the error bars are calculated using the standard deviation. (**c**) Change of the NiPAAm:AAm gel diameter in water when the temperature is cycled between 37 °C and 45 °C, showing that NiPAAm gels can be thermally actuated for several cycles between 37–45 °C. (**d**) Percentage of TRITC-dextran (20 kDa) released from a device immersed in water when thermally actuated (◻) at 45 °C for 15 minutes once per day for four consecutive days, or not actuated (■). All devices were kept at 37 °C prior to and between actuations. Fluorescence (λ_ex_ = 540 nm, λ_em_ = 580 nm) was measured using a plate reader as a function of time. All data points correspond to the average of at least 3 different experiments and the error bars are calculated using the standard deviation. (**E**) Release of different MW fluorescent dextran from capsule over time.

Above its critical temperature, NiPAAm gels contract; this transition temperature of the gel can be tuned by changing the concentration of the monomer (AAm) and crosslinker (MBAAM). By increasing the concentration of AAm, we achieved a gel composition that contracted ~55% between 37 °C and 55 °C, comparable to other systems^24^ (Fig. 2a). Also, by increasing the amount of crosslinker, we could lower the temperature at which the gel starts to contract (Fig. 2a). However, we found an excessively high percentage of crosslinker resulted in a brittle gel, whereas a low percentage resulted in an excessively soft gel that was difficult to manipulate. For subsequent capsules, we chose a gel composition of 85:15 w/v NiPAAm:AAm; when heated from 37 °C to 50 °C, this gel contracted to 36% of its original volume, with 20% of this volume change taking place between 37 °C and 45 °C, and less than <8% for changes between 37 °C and 42 °C). (The small contraction of the gel for temperatures under 40 °C ensured that the device will not be actuated in cases of mild to high fever). This result was reproducible in saline solution to mimic physiological conditions (Fig. 2b). We further tested the suitability of this gel composition for reversible shrinking and swelling due to temperature changes. The NiPAAm-based gels shrank and swelled for six transitions in temperature between 37 °C and 45 °C over 15 minutes with reproducible dimensions (Fig. 2c). (Previous models demonstrated 45 °C can be sustained at the FUS focal point for at least 15 min without inducing damage to neighboring cells^25^). More details regarding the characterization of the gel contraction with temperature can be found in Supporting Information.

In addition to changes in gel volume, we studied the release of TRITC-dextran (20 kDa) by immersing dye-loaded gels contained in PDMS capsules in a 37 °C water bath and measuring fluorescence of the surrounding media. Over 4 days, very little dextran leaked out of the capsule (<1.5%; most of it within the first 45 minutes); this background volume can be decreased in the future with improved design of the capsule. With each transfer to a hot-water bath (45 °C), the gels rapidly contracted and released ~7% of its dye to the surrounding media (Fig. 2d). Moreover, we assessed the ability of the capsule to hold and release TRITC-dextran of different molecular weights (4.4, 20, 65–85 and 155 kDa) corresponding to a range of hydrophilic drugs from small proteins to antibodies. We loaded up to 120 micrograms of compounds inside the capsule, equivalent to the masses of pharmacological drugs in other local controlled-release devices^26^,^27^,^28^. At 37 °C, very little TRITC-dextran was released (<2%; Fig. 2e); when the temperature changed transiently to 45 °C, the amount of dextran released was inversely related to the dextran molecular weight.

### FUS-triggered change in local temperature and compound release

**Figure 3:**
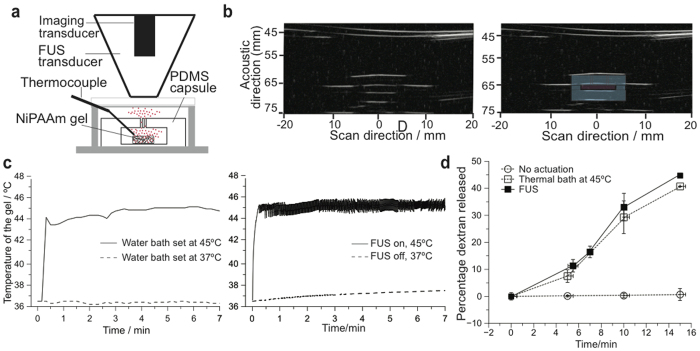
FUS-triggered local temperature change and compound release of implanted gel. (**a**) Schematic diagram of the setup for testing FUS-triggered drug release. (**b**) Ultrasound image of the device. The image shows a perpendicular cut of the device in the x-z plane. For clarity, a drawn overlay of the device shows its location. (**c**) Representative trace of a temperature profile inside the NiPAAm-co-AAm gel by immersing the device in a thermal bath, and applying FUS. The FUS was controlled using the temperature reading from a thermocouple inserted in the gel. For all experiments, the starting temperature was 37 °C. (**d**) Release of TRITC-dextran (20 kDa) as percentage release from the device when actuated (thermally (◻) or by FUS (■)), or no actuation applied (○). FUS-induced heating was able to mimic gel contraction and release produced by submersing the gel in 45 °C water bath. Temperatures of 37 °C (for no actuation) and 45 °C (for thermal bath and FUS) were verified by a temperature probe. All data points correspond to the average of at least 3 different experiments and the error bars represent the standard deviation.

We integrated a spherically sectioned FUS transducer (aperture radius of 40 mm, focal length of 90 mm) with an imaging transducer (focal length of 60 mm, center frequency of 7.5 MHz, −6-dB bandwidth of 4 MHz) (Fig. 3a). This setup could be used for both ultrasonically imaging (Fig. 3b) and triggering the hydrogel device. For imaging, the capsule can be identified from a structured pattern (from ultrasound echos reflected from layers of the device) surrounded by a black void (as fluid in the test chamber does not scatter back any ultrasound) (Fig. 3b). For triggering, the application of 1.5-MHz FUS was continuously adjusted using temperature readings from thin-wire thermocouples placed inside the capsule (Fig. 3a); this feedback loop helped maintain the local temperature at the desired set point (see Supplementary Online Information for more details). The transducer driving voltage was chosen to produce moderate focal intensity, which was less than 500 W/cm^2^ as estimated using a radiation-force balance. The acoustic impedance of PDMS was well-matched to tissue and water so that sufficient energy penetrated into the device, resulting in local heating from 37 °C to 45 °C (Fig. 3c). We found that FUS triggering elevated the gel temperature to the set point of 45 °C within 33 ± 19 s (n = 10), compared to 3 ± 1 min by immersing the gel in a warm-water bath. Moreover, FUS-induced heating was successful in releasing a similar percentage of dextran mass as when actuated by a thermal bath (Fig. 3d).

### Characterization of temperature change around capsule by simulation and experiment

**Figure 4:**
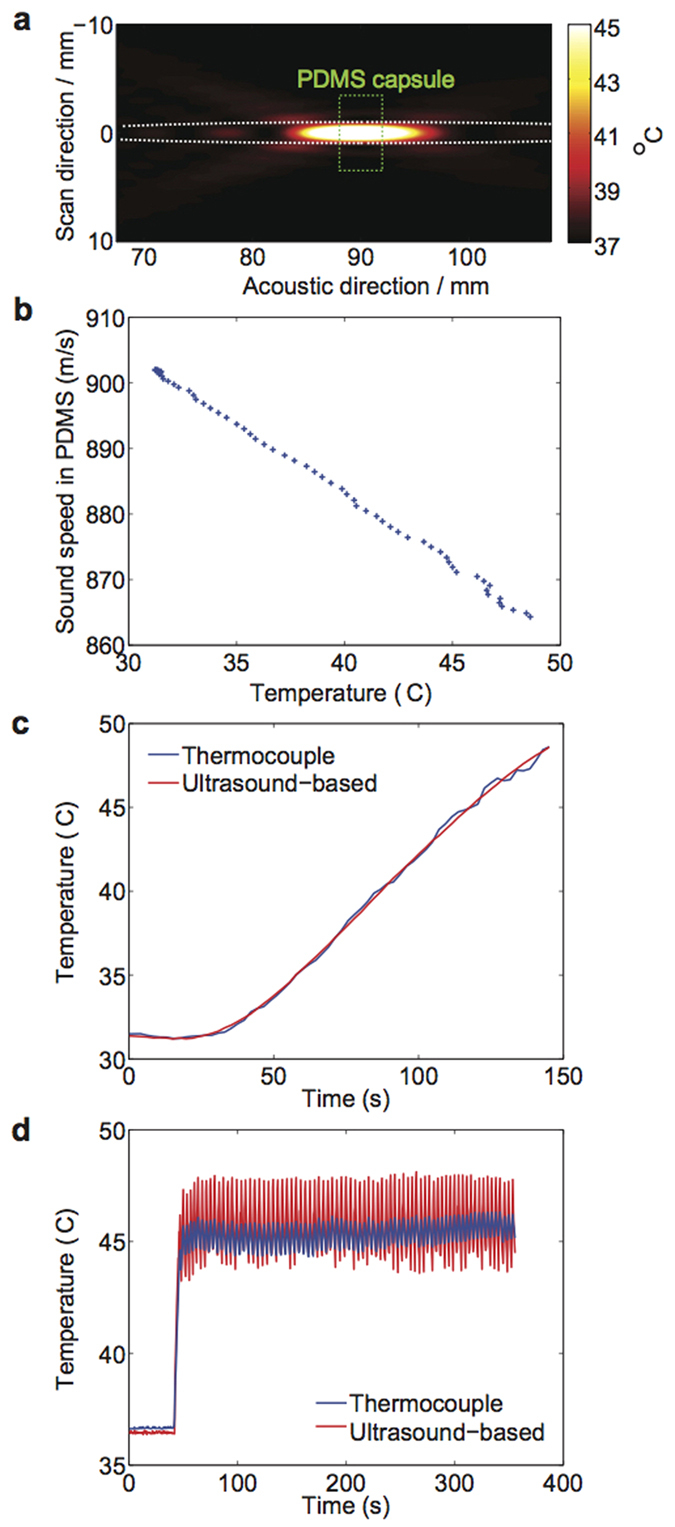
Simulation and characterization of local FUS-induced temperature change. (**a**) Simulation of FUS-induced heating in tissue. Region within black dash represents the FUS focal region, and region within white dash represents −6-dB beamwidth of the A-mode transducers. The gel device is shown within green dashes. (**b**) Experimental validation of ultrasound-based thermometry. Values of sound speed were measured for temperatures from 33 to 50 °C. Consecutive pulse-echo measurements (M-scan) can provide a measure of the apparent strain in the 1D image, which corresponds to thermally induced change in arrival times of echoes from the top and bottom of the PDMS layer. (**c**) Temperature-time profile of PDMS disks conductively heated by a hot plate, as measured by ultrasound thermometry and a thermocouple. (**d**) Use of ultrasound thermometry as feedback to guide FUS triggering. A sustained temperature was achieved over time, as measured by a thermocouple (blue).

To further characterize the spatial extent of temperature changes around the capsule, we simulated the continuous-wave pressure field generated by the FUS transducer using the fast nearfield method^29^,^30^ (see Supplementary Online Materials and Fig. S1), and predicted the resulting temperature distribution using Penne’s Bioheat equation^31^. This simulation assumed constant acoustic power at the FUS focal region, and thermal conductivity of surrounding tissue approximating physiological values of well-vascularized tissues. The results showed that the FUS-heated region was contained within the sensing volume of the A-mode imaging transducer (Fig. 4a, white dashes). This result confirmed that the co-axial FUS and A-mode transducers were well-suited for real-time thermometry during FUS-induced local heating of gel devices.

**Figure 5:**
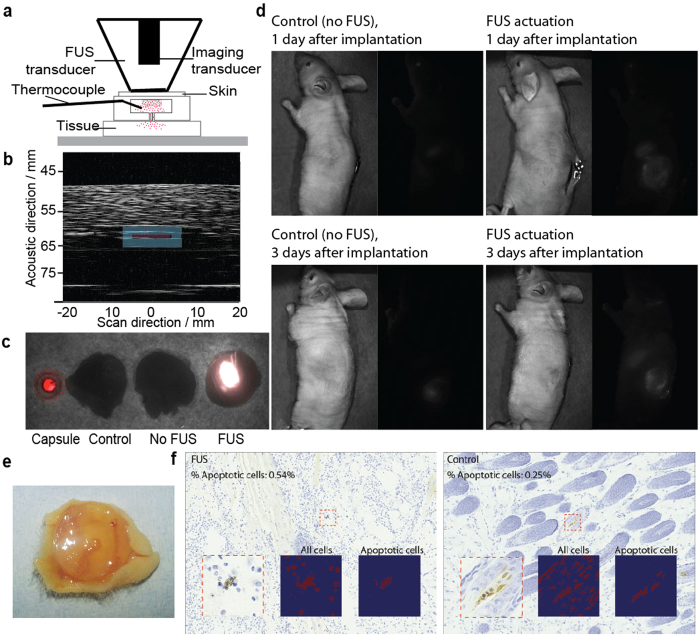
Compound release in *ex vivo* chicken and *in vivo* mouse. (**a**) Schematic diagram of the experiment set up used for the FUS-induced *ex vivo* experiments. During the *ex vivo* experiments the imaging transducer was used to non-invasively monitor the temperature inside the gel to control the FUS using a feedback loop. (**b**) Ultrasound image of the device placed between two layers of 10 mm of chicken breast. The image shows a perpendicular cut of the device in the x-z plane. For clarity, a drawn overlay of the device shows its location. (**c**) Merged bright image and fluorescent image (λex = 670 nm, λem = 702 nm) of a gel capsule sandwiched between a lower layer of chicken tissue and a top layer of mouse skin. The four samples were capsule alone, chicken tissue alone, capsule embedded in tissue at 37 °C on a hot plate for 10 minutes, and FUS-actuated (at set-temperature of 45 °C) for 10 minutes. The ultrasound actuation took place from the top through the mouse skin and the AlexaFluor-dextran release was from the bottom part of the capsule towards the chicken tissue. (**d**) *In vivo* results for FUS induced dextran release. Representative bright field and fluorescence images of control (no FUS actuation) and FUS-administered mouse. All mice were sacrificed and NiPAAm gels explanted before imaging. FUS actuation was for all cases 10 minutes at <00 W/cm^2^ (estimated using radiation-force balance as demonstrated in prior studies^67^,^68^). (**e**) Photograph of an explanted device after 2 weeks of being implanted in a mouse. (**f**) TUNEL stained histology samples of the tissue immediately on top of the gel for a mouse treated with FUS and a control one (no FUS).

Moreover, our experiments confirmed that our capsule material (i.e. PDMS) gives rise to a linear and predictable relationship between sound speed and temperature^32^,^33^ (Fig. 4b). This result suggests we can reliably infer the temperature of the gel-based device and to monitor its actuation from the measured pulse-echo signals (even though sound speed is a non-monotonic function of temperature in tissue *in vivo*^34^). To further confirm this capability, we measured the temperature-time profile of a PDMS disk being heated on a hotplate, and found that the ultrasound-based measurements agreed with the reading from a thermocouple inserted into the PDMS disk (Fig. 4c). In a final setup, we used sound speed-based temperature measurements as real-time feedback to pulse-width modulate the FUS transducer in order to sustain a set temperature of 45 °C in a gel capsule placed between a top 5-mm layer of mouse skin and a bottom piece of 3-mm chicken tissue (Fig. 5a). We took advantage of the observation that pulse-echo signals corresponding to the interfaces of PDMS capsules can be easily identified, even when embedded in a tissue. This real-time feedback of temperature as measured from sound speeds (red trace in Fig. 4d) enabled the gel temperature to be automatically sustained at 45 °C ± 0.5 °C for 10 minutes, as confirmed by a thermocouple embedded in the NiPAAm gel (blue trace in Fig. 4d).

### FUS-triggered compound release in tissue

To test the ability to trigger gels embedded in a tissue, we first embedded a gel capsule between a layer of mouse skin (top, 5-mm) and chicken tissue (bottom, 3-mm) (Fig. 5a). Here, the PDMS capsules contained NiPAAm gel with 10 kDa AlexaFluor 680 labeled dextran, and had two openings of 160 μm diameters (where the fluorescent molecule can diffuse out of the capsule) facing down on the bottom layer of tissue (AlexaFluor 680 labeled dextran was chosen to minimize the effects of tissue auto-fluorescence during imaging). First, we mechanically scanned the transducer assembly across the tissue while acquiring pulse-echo signals (Fig. 5b). Because the ultrasound images of tissue are rich in speckle, the capsule can be identified from the structured pattern and absence of speckle. In addition, we detected release of the fluorescent tracer after FUS triggering, but not in a control tissue without FUS triggering (Fig. 5c).

### FUS-triggered compound release in mice

Next, we investigated the feasibility of employing FUS to trigger release of fluorescent dextran from the gel capsules *in vivo.* The devices were surgically implanted subcutaneously at the dorsum of four pairs of mice. We chose a subcutaneous model, which is a site commonly used in mouse tumor models^35^,^36^,^37^ and is experimentally amenable to surgical retrieval of capsule after applying FUS experiments. Briefly, the procedure was as follows: we anesthetized the mice (either 1 day or 3 days after implantation of the capsule), manually aligned the FUS transducer over the device, applied an acoustic-coupling gel between the transducer and the mouse skin (to facilitate introduction of acoustic energy into the tissue), and employed ultrasound-based thermometry as feedback to elevate and maintain the temperature of the implanted gel capsule at 45 °C for 5 minutes. We sacrificed the mice immediately afterwards, surgically removed the capsule (so that the released molecules could be visualized without the high background of the gel), and visualized the fluorescence of the mice with a small-animal fluorescence imager. Brightfield and fluorescence images of the tissue surrounding the capsules showed an increase in fluorescent signal in mice administered with FUS compared to those without FUS (Fig. 5d).

Finally, we examined damage to surrounding tissue upon FUS triggering. Conventional applications of FUS therapy typically involve target temperatures between 50 to 80 °C, which are achieved without causing tissue damage^38^,^39^. According to a model from Sapareto and Dewey, an equivalent thermal dose our FUS treatment parameters (45 °C for 5 min) was 43 °C for 20 min, which did not produce significant tissue damage^40^. Here, we implanted our capsule (without fluorescent dextran to avoid interference with subsequent histological staining) subcutaneously into the dorsum of mice; applied FUS actuation after two weeks, and immediately afterwards explanted the surrounding tissue (including skin and fascia layer). (Tissue sections were also obtained from control mice 1, 2 or 4 weeks after implantation of device, but with no FUS actuation). Examination of the explanted gels indicated no signs of structural damages to the capsule (Fig. 4e) or fibrous capsule formation around the device. Furthermore, gel implantation did not result in chronic inflammation or necrosis of the surrounding tissue as indicated by H&E stain and trichrome stain, respectively (Fig. S2). We analyzed tissue sections for apoptosis (to discard thermal damage to the iTAG surrounding tissue) using detection of single and double stranded DNA breaks (Fig. 4e) (i.e. using a TUNEL kit, as performed previously^41^). The percentage of apoptotic cells was low (<1%) for both the FUS-exposed mouse and the control mouse. These results confirm that if thermal damage exists (unlikely based in the previous results), it will be limited to the diseased tissue and not the healthy tissue.

## Discussion

We demonstrated a novel strategy for delivering compounds *in vivo* which integrated: 1) an implantable capsule containing a drug-loaded gel; 2) an imaging and actuation instrument based on focused ultrasound; and 3) a control feedback process which uses temperature measurements based on PDMS sound speed to achieve controlled changes in temperature and release of compounds. Compared to previous methods for using FUS for drug delivery, the iTAG strategy could alleviate challenges associated with finite lifetime of drug-carrying agents in circulation, and difficulties achieving cytotoxic levels due to relatively low loading capacity of liposomes and microbubbles^17^. Also, previous ultrasound-based release methods often exhibited inertial cavitation, which posed problems when used *in vivo*^42^. Mechanical index, which is an empirically determined safety-factor that describes the risk of cavitation associated with pulsed (2–3 cycles) diagnostic ultrasound^43^, is not sufficient for definitively confirming presence or absence of cavitation, particularly in a therapeutic application involving quasi-continuous-wave ultrasound. Therefore, we employed passive cavitation detection by means of the co-axial A-mode transducer as demonstrated in prior works^34^,^35^. Our A-mode transducer detected no broadband noise in the pulse-echo signals and confirmed an absence of inertial cavitation^32^,^33^. Furthermore, the A-mode transducer did not detect an increase in backscattered HIFU harmonics, which indicated that the relatively low-power FUS employed in this study did not generate any stable cavitation^34^. Therefore, currently the iTAG method relies on purely thermal effects generated by FUS. In the future, the iTAG method can potentially integrate other FUS capabilities, such as enhancement of vascular and cell permeability (in order to increase diffusion of drug through diseased tissues)^44^, or be integrated with magnetic resonance^45^,^46^ to facilitate implants of the capsule in anatomical sites such as the brain. When implementing this approach at deep anatomical sites, higher FUS power than currently employed might be necessary to overcome tissue attenuation. In these scenarios, large FUS arrays (effective F-number <1) that can precisely focus ultrasonic energy at the desired location (i.e., the implanted device) while maintaining low power densities in the intervening tissue^43^,^44^ should be employed to avoid collateral damage associated with temperature elevation or cavitation in off-target locations. While it is possible that this approach might produce mildly hyperthermic temperatures (between 42–45 °C) in normal tissue adjacent to the tumor, previous studies including several patient studies have shown that hyperthermic cytotoxicity is largely constrained to the tumor and the normal tissue is unaffected^47^. Mild hyperthermia (<45 °C) has been demonstrated to significantly enhance the therapeutic impact of adjuvant treatments in a variety of anatomical sites in clinical studies and is well-tolerated by patients including those in advanced stages of cancer^47^,^48^,^49^,^50^.

The iTAG strategy complements implantable devices published previously. For example, implantable capsules with wireless control have been successfully used for capsule endoscopy and biopsy^51^,^52^; to be used outside the gut, however, such devices must be re-designed to increase biocompatibility and decrease size. Compared to recently developed drug-releasing microchips^6^, iTAG requires no battery or intrinsic power supply, hence circumventing limitations of toxicity of battery or electronic components. FUS has a large penetration depth through tissue (>10 cm)^40^, compared to some other methods for controlling drug delivery^53^,^54^,^55^. A recent study used ultrasound to disrupt calcium-induced crosslinking of alginate gel and to trigger drug release^56^. Compared to this work and other designs with direct contact between drug-carrying gel and external tissue^9^,^24^, iTAG features a gel encapsulated inside a capsule to prevent cell invasion and minimize background leakage. In addition, the use of a thermally-sensitive gel obviates the need for additional chemical agents for crosslinking or uncrosslinking, which could interfere with the robustness of the triggering as the agent diffuses out of the gel over time. Continuing work will substitute the PDMS capsule for more biocompatible/biodegradable materials^57^,^58^,^59^,^60^,^61^ such as PEG-based gels. Use of a degradable material will avoid the need of explantation of the device after treatment.

An implantable compound-delivering capsule and triggering system – especially if it is cost-effective, portable, and does not require large equipment – could be attractive in clinical practice. As the concepts of personalized medicine^1^ and individualized medicine^62^ take hold, a therapeutic dose could be tuned in real time in response to a patient’s health (for implantable drug-releasing devices, tailored dosing is also attractive in addressing patient-to-patient variations in anatomy and surgical procedure^2^,^3^). Specific clinical applications include local release of chemotherapeutic drug (avoiding systemic toxicity) and local release of hormones^6^. Before clinical use, more work on the current version of iTAG would need to be done: for example, to match the drug-loading capacity and expected lifetime of the capsule to the desired application, possibly by integrating multiple implants or larger implants with multiple reservoirs. In a patient setting, iTAG can leverage the already widespread use of ultrasound in outpatient settings for imaging biopsies^63^ as well as interventions such as thermal ablation by radio frequency^64^ and FUS^39^,^65^.

## Methods

Details on characterization of the capsule *in-vitro*, FUS experimental setup, simulations, FUS triggering, and histology are in Supporting Information Online.

### Reagents

N-isopropylacrylamide (NiPAAm), acrylamide (AAm), N, N-methylenebisacrylamide (MBAAm) and TRITC-dextran (20, 65–85 and 155 kDa) were purchased from Sigma-Aldrich. AlexaFluor-dextran 680 (10 kDa) was purchased from Invitrogen. The photoinitiator 2-hydroxy-2-methyl-1-phenyl-propan-1-one (Darocur-1173) was purchased from Ciba® and polydimethylsiloxane (PDMS) Sylgar 184 from Dow Corning®. All aqueous solutions were prepared using deionized water. All chemicals were of analytical reagent grade and were used as received without any further purification.

### Fabrication of the gel and capsule

NiPAAm-co-AAm gels were fabricated by UV photo-polymerization. A schematic of the fabrication process is shown in Fig. 1. Briefly, the pre-polymer solution mixture, containing NiPAAm (1.7 M, 20% w/w), the co-monomer AAm (85:15 molar NiPAAm:AAm), the crosslinker MBAAm (5% w/w respect NiPAAm monomer) and the photoinitiator Daracour 1173 ® (0.1% w/w) dissolved in water:ethanol (50:50 v/v), was placed inside a PDMS container and covered with a glass slide. A photo-mask transparency with the desired design was placed over the glass slide and the photo-polymerization was initiated by irradiating with UV light (350 nm) (Omnicure series 2000, Lumen Dynamics Group Inc., Canada). The polymerization was completed within 48 seconds resulting gels, that in water at room temperature, were discs of 7.2 ± 0.08 mm in diameter and 1.55 ± 0.06 mm in thickness. Then, the hydrogels were rinsed thoroughly with water to remove the non-crosslinked pre-polymer and allowed to swell to equilibrium at room temperature in deionized water for 24 h.

To load the fluorophore labeled dextran inside the gels, first the gels were dried at room temperature for 24 h. Next, the dry gels were allowed to swell in a concentrated solution of dextran (200 μM) for a day. Finally, the loaded-gels were dried again for 48 h and kept in the fridge until use. In order to minimize the diffusion of the dextran from inside the NiPAAm:AAm gel to the surrounding media, and have a better control over the pulsatile release, the gels were encapsulated inside a containers made of PDMS^66^. The latter were designed to exactly fit the dimensions of the gels at 37 °C (6.6 ± 0.07 mm in diameter and 1.5 ± 0.08 in thickness in water and 5.2 ± 0.1 mm in diameter and 1.3 ± 0.07 in PBS buffer); and therefore minimize non-desired dextran leaks. The PDMS capsules were fabricated by replica molding of a polyacrylamide master. First, the PDMS elastomer base and the curing agent were mixed at ratio 10:1 w/w and poured over the master. After degassing the solution in a vacuum chamber the PDMS was cured at 70 °C for 30 min. Then, the cured PDMS was peeled-off from the master, and the dried loaded-NiPAAm-co-AAm gel placed inside the cavity and irreversibly bonded to a PDMS coverlid using oxygen plasma. The PDMS coverlid was pierced twice with a 30-gauge needle before the bonding step to open the two apertures of the capsule. After the bonding the capsule was filled with deionized water or PBS buffer using a syringe and in less than 30 minutes the NiPAAm-co-AAm gels swollen to occupy the entire chamber volume blocking the releasing holes.

Animal experiments. One sterile device was implanted subcutaneously in the dorsum of 8 male athymic nude mice (NCr nude, Taconic) under isoflurane anesthesia. The incision (≈2 cm) was closed using surgical staples and liquid pockets were created around the device injecting PBS saline buffer. During FUS actuation and *in-vivo* imaging, the mice were anaesthetized using isoflurane, so the animals did not have to be restrained. All animals were fed an alfalfa-free diet (Teklad Global Diet, 2914) for at least a week before surgery to minimize autofluorescent signals from the digestive track in the far-red range during *in vivo* imaging. All animal procedures were approved by Columbia University Animal Care and Use Committee (protocol AC-AAAG1300), and were carried out in accordance with the approved guidelines.

## Additional Information

**How to cite this article**: Ordeig, O. *et al.* An implantable compound-releasing capsule triggered on demand by ultrasound. *Sci. Rep.* **6**, 22803; doi: 10.1038/srep22803 (2016).

## Supplementary Material

Supporting Information
