# Supplementary material for: An implantable compound-releasing capsule triggered on demand by ultrasound
Source: Sci Rep. 2016 Mar 11;6:22803. doi: 10.1038/srep22803 (PMC4786798; doi:10.1038/srep22803)
Supplement: Supporting Information [file srep22803-s1.pdf]

Supporting Information for:

An implantable compound-releasing capsule triggered on demand by ultrasound

Olga Ordeig[+1], Sau Yin Chin[1], Sohyun Kim[1], Parag V. Chitnis[+2,3*] and Samuel K. Sia[1*]

[1] Department of Biomedical Engineering, Columbia University, 351 Engineering Terrace, 1210 Amsterdam Avenue, New York, NY 10027, United States.

[2]Department of Bioengineering, George Mason University, 4400 University Drive, Fairfax, VA 22032, United States.

[3]F. L. Lizzi Center for Biomedical Engineering, Riverside Research, New York, NY 10038, United States.

Materials

N-isopropylacrylamide (NiPAAm), acrylamide (AAm), N, N-methylenebisacrylamide (MBAAm) and TRITC-dextran (20, 65-85 and 155 kDa) were purchased from Sigma-Aldrich. AlexaFluor-dextran 680 (10kDa) was purchased from Invitrogen. The photoinitiator 2-hydroxy-2-methyl-1-phenyl-propan-1-one (Darocur-1173) was purchased from Ciba® and polydimethylsiloxane (PDMS) Sylgar 184 from Dow Corning®. All aqueous solutions were prepared using deionized water. All chemicals were of analytical reagent grade and were used as received without any further purification.

Fabrication of gel and capsule

NiPAAm-co-AAm gels were fabricated by UV photo-polymerization. An schematic of the fabrication process is shown in Figure 1. Briefly, the pre-polymer solution mixture, containing NiPAAm (1.7 M, 20% w/w), the co-monomer AAm (85:15 molar NiPAAm:AAm), the crosslinker MBAAm (5 % w/w respect NiPAAm monomer) and the photoinitiator Daracour 1173 ® (0.1 % w/w) dissolved in water:ethanol (50:50 v/v), was placed inside a PDMS container and covered with a glass slide. A photo-mask transparency with the desired design was placed over the glass slide and the photo-polymerization was initiated by irradiating with UV light (350 nm) (Omnicure series 2000, Lumen Dynamics Group Inc., Canada). The polymerization was completed within 48 seconds resulting gels, that in water at room temperature, were discs of 7.2 ± 0.08 mm in diameter and 1.55 ± 0.06 mm in thickness. Then, the hydrogels were rinsed thoroughly with water to remove the non-

crosslinked pre-polymer and allowed to swell to equilibrium at room temperature in deionized water for 24h.

To load the fluorophore labeled dextran inside the gels, first the gels were dried at room temperature for 24 h. Next, the dry gels were allowed to swell in a concentrated solution of dextran (200 μM) for a day. Finally, the loaded-gels were dried again for 48 h and kept in the fridge until use. In order to minimize the diffusion of the dextran from inside the NiPAAm:AAm gel to the surrounding media, and have a better control over the pulsatile release, the gels were encapsulated inside a containers made of PDMS [16]. The latter were designed to exactly fit the dimensions of the gels at 37ºC (6.6 ± 0.07 mm in diameter and 1.5 ± 0.08 in thickness in water and 5.2 ± 0.1 mm in diameter and 1.3 ± 0.07 in PBS buffer); and therefore minimize non-desired dextran leaks. The PDMS capsules were fabricated by replica molding of a polyacrylamide master. First, the PDMS elastomer base and the curing agent were mixed at ratio 10:1 w/w and poured over the master. After degassing the solution in a vacuum chamber the PDMS was cured at 70ºC for 30 min. Then, the cured PDMS was peeled-off from the master, and the dried loaded-NiPAAm-co-AAm gel placed inside the cavity and irreversibly bonded to a PDMS coverlid using oxygen plasma. The PDMS coverlid was pierced twice with a 30-gauge needle before the bonding step to open the two apertures of the capsule. After the bonding the capsule was filled with deionized water or PBS buffer using a syringe and in less than 30 minutes the NiPAAm-co-AAm gels swollen to occupy the entire chamber volume blocking the releasing holes.

Characterization of gel contraction with temperature

Gels of different compositions (NiPAAm:AAM ratios ranging from 100:0 to 80:20 and amount of crosslinked, MBAAB, ranging from 5% to 15%) were fabricated as described above; and its thermal response characterized over a range of temperature from 20ºC to 50ºC. Each gel was immersed for at least 60 minutes (to ensure equilibrium) in a water bath at different temperature set points. Then, gels were carefully transferred to a microscope slide and an optical image was taken using an optical microscope with an integrated camera. The gel dimensions were measured using a measuring tool from the microscope software. The change of gel dimensions with temperature was represented as a relative diameter change to its dimensions at room temperature (see Figure 2a and 2b).

Quantification of gel loading capacity

Gels were loaded by swelling from its dry form in a concentrated solution of dextran (200 μM). To quantify the amount of labeled dextran loaded in the gel, the latter was immersed in a clean water bath (1 mL) and the dextran was allowed to diffuse out form the gel matrix. Every 2 hours the gel was transferred to a clean water bath and the levels of dextran released from the gel quantified by fluorescence. This process was repeated until no dextran was detected in the baths. The sum of the amount of dextran released in all the baths corresponds to the amount of dextran loaded in the gel.

Figure 2e summaries the amount of dextran loaded per gel using different molecular weights of dextran.

Characterization of gel capsules *in vitro*

For contraction of the NiPAAM-co-AAM gels using a conventional thermal bath, the devices loaded with 54.7 ± 6.8 μg TRITC-dextran (20kDa) were individually immersed in small containers filled with 3 mL of deionized water at 37ºC for at least 45 minutes. To contract the gel, the device was transferred to a second container set at 45ºC. Every 3 to 5 minutes, 200 μL of water were removed from the container to quantify the amount of TRITC-dextran released and replaced with 200 μL of fresh water. The water baths and the gels temperature were monitored in real time using two thermocouples (HYPO-33-1-T-G-60-SMPW-M, Omega) connected to a digital thermometer (HH506RA, Omega). The total amount of dextran released was quantified by analyzing the different water bath solution with fluorescence.

For the FUS-induced release experiments, we used a high-intensity ultrasound spherically sectioned transducer with a center frequency 1.525 MHz, focal length of 90 mm, and an aperture radius of 40 mm. The FUS transducer also had a central aperture containing a coaxial diagnostic A-scan transducer (center frequency 7.5MHz, focal length 60 mm). The foci of the two transducers were co-aligned. The diagnostic transducer was used to produce B-mode images of the gels and provide guidance to align the FUS focal point into the gel (see *Figure 2-c*). A plastic truncated-cone shell was attached to the transducer, filled with degassed water and its end covered with a thin membrane. The truncated end of the cone was approximately 25 mm less than the radius of curvature of the transducer, i.e., the acoustic energy was focused 25 mm in front of the truncated cone. A thick piece of gel phantom (Aqualfex, Parker Laboratories) was used to adjust the focal distance and facilitate transmission of acoustic energy. Acoustic-coupling gel was also used to provide a good coupling during the scans.

Although originally the FUS transducer was designed for thermal therapy using high-intensities (HIFU), for these experiments it was operated at moderate intensities (<500 W/cm^2). This transducer was driven by a function generator (Model 33250A, Aglient) in combination with a RF power amplifier (Model A150, ENI). For our experiments, the pre-amplified driving voltage ranged between 70 and 300 mV. The focal acoustic intensity of the transducer was characterized using radiation-force-balance method described in previous studies [PC6,PC7]. This method relies of measurement of acoustically induced force acting on an sound-absorbing target placed on a scale. We operated the transducer in the third harmonic mode to measure the acoustically exerted force on a sound-absorbing target placed on a scale. The precision of the scale was not adequate for reproducible and accurate measurements of acoustically induced force when the transducer was operated at driving voltages below 400 mV. We measured the intensity over a wide range (100-1000 mV) to include regime where our setup provided reliable measurements. Force measurements acquired using the scale were then used to estimate the focal intensities ($P = 2Fc/(cos(\alpha_1)+cos(\alpha_2))/DF$; $I = P/A$) at each driving voltage. P is acoustic power, F is measured force, c is sound speed, α_1 and α_2 are half angles corresponding to transducer hole and diameter, respectively, DF is the duty factor, I is intensity, and A is cross-sectional area of the FUS beam along the focal plane. The intensity values were divided by nine to obtain estimates of focal intensity produced by the transducer when operating at the fundamental frequency (Fig. S1). Based on these measurements, the focal intensities necessary to thermally actuate the hydrogel-based devices were below 500 W/cm^2.

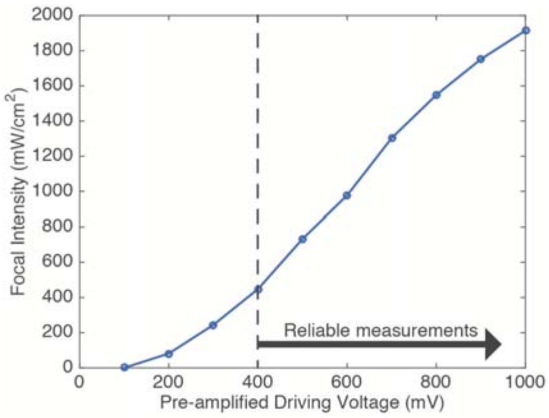

Figure S1. Focal acoustic intensity of the FUS transducer estimated using a radiation-force balance.

For the FUS experiments, the PDMS capsule (containing the loaded gel) was fixed at the bottom of a bigger PDMS container filled with deionized water at 37ºC. The PDMS container was then covered with a thin layer of PDMS (~0.5 mm), a 1.5 mm thick gel phantom (Aquaflex, Parker Laboratories) and acoustic-coupling gel. The application of FUS resulted in local heating from 37ºC to 45ºC, inducing the release of dextran from the gel. The temperature in the gel was sustained to 45 ± 0.5ºC for all the duration of the experiment (between 2 and 15 minutes) by turning the FUS 'on' and 'off' using a temperature reading feedback loop from a thermocouple inserted in the gel. The feedback loop was implemented using a PC-based instrumentation-control software, which recorded and monitored the thermocouple temperature and also controlled the function generator used for driving the FUS transducer. The control software turned off the signal output of the function generator when the *in situ* temperature exceeded 45.5ºC. Conversely, the control software turned the signal output back on when the temperature decreased to a value less than 44.5ºC. At the end of each experiment, the FUS was turned 'off' and the temperature in the gel immediately dropped from 45ºC back to 37ºC stopping the FITC-dextran release. The temperature in the hot plate set at 37ºC was stable for 10 minutes at 37.3 ± 0.3ºC (data not shown).

For all *in vitro* experiments, the amount of TRITC-dextran released from the gel to the surrounding media was analyzed by fluorescence (λ_{ex} = 540 nm, λ_{em} = 580 nm) using a plate reader (Cinergy 4, Biotech). TRITC-dextran concentrations were calculated in accordance to pre-established calibration curves (data not shown).

Simulation of FUS-induced heating

An open source package for linear simulation of ultrasound propagation in tissue, Fast Object- oriented C++ Ultrasound Simulator (FOCUS) [1, 2], was employed to calculate the acoustic-pressure field generated by the FUS transducer when operated at 1.5 MHz. The primary goal of the simulation study was to verify that the fixed-focus FUS transducer could provide localized heating in a region that corresponded to the location and the size of our devices. Due to the axi-symmetric nature of the acoustic field produced by a spherically sectioned transducer, acoustic-pressure calculations were performed on a 2D grid that was 20 mm in the scan direction and 40 mm in the acoustic direction and centered around the focus of the FUS transducer (denoted by the green * in Fig. S1). Inter-nodal spacing in the computation grid was $\lambda/12$, where λ was the acoustic wavelength. FOCUS discretizes the acoustic source, i.e., the FUS transducer in this case, into apodized rectangles and employs super-position of time-harmonic numerical calculations of the pressure response from individual rectangular pistons.

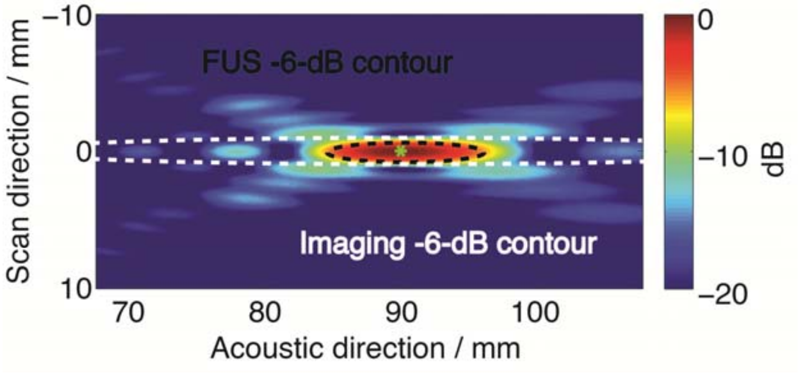

Figure S2. Simulation of spatial distribution of acoustic power delivered to tissue using the FUS transducer.

The pressure field *p(r)* was then used as input to determine the spatial distribution of the acoustic power delivered to the tissue, which is given by the following equation:

$$Q(r) = \frac{\alpha}{\rho c} \sum p(r,t) p^{*}(r,t), \tag{1}$$

where α is absorption coefficient, ρ and c represent the density and sound speed of the medium, respectively, and the superscript * represents conjugate transpose. The acoustic power deposition was used as an input for the steady-state bio-heat equation given by

$$K\nabla^2 T(r) - W_b C_b T(r) + Q(r) = 0, \tag{2}$$

where K is the thermal conductivity of tissue, C_b is the specific heat of blood, W_b is the blood perfusion rate, T is the temperature increase in tissue. In this equation, the first and the third terms describe heat accumulation at any point in the computation grid resulting from thermal conduction and acoustic-energy deposition, respectively. The second term describes the convective heat loss (an effective heat-sink) associated with blood perfusion. Equation 2 is solved with an iterative finite-difference scheme to simulate FUS-induced heating and its solution (*T(r)*) predicts the temperature distribution resulting from sonication. FOCUS allows user to input the amplitude of the acoustic pressure at the transducer face. This value was

chosen such that a lateral extent of 45°C region was achieved in a region that was 1-mm wide in the scan direction. The tissue volume that reached 45°C was contained within the NiPAAm device (Fig. 4A, green dashes) in the lateral (scan) direction but exceeded the device dimension in the axial (acoustic) direction.

PDMS-only disks. Disks made only of PDMS were studied to show the ultrasound thermometry was based only on PDMS.

Ultrasound thermometry. For thermometry, because the thermocouple was oriented orthogonally to this cylinder, careful alignment of the thermocouple with the focus of the FUS transducer was critical to accurate temperature assessment. In some instances, errors in aligning the focus of the FUS transducer with the thermocouple tip resulted in a thermocouple reading that was lower than the true temperature at the focus and consequently, more time was needed for thermocouple to reach the set-point temperature. To achieve a good alignment, the thermocouple was always inserted to the center of the gel and B-mode ultrasound images of the device were used to align the FUS focal volume to the center of the gel. For all experiments that required more than 60 seconds to reach 45ºC at the thermocouple tip, the FUS transducer was considered to be misaligned with the thermocouple the results were excluded.

FUS triggering in tissue

For the *ex vivo* tissue experiments we used the same FUS transducer described above with the only difference that a longer water-filled conical section was mounted to its housing (to couple ultrasonic energy into hydrogel structures or tissue). The end of this cone was 5 mm less than the transducer focal distance, so a gel placed under a 5 mm layer of tissue was

perfectly placed at the FUS focal point; eliminating the need of using gel phantoms or Aquaflex pads to adjust the focal distance. Acoustic-coupling gel was used to facilitate transmission of acoustic energy into the tissue.

To add an imaging modality, the ultrasound apparatus shown in Figure 3-a also included an A-mode imaging transducer co-axially located in the center hole of the FUS transducer. This transducer had a focal length of 60 mm, a center frequency of 7.5 MHz, and a bandwidth of 4 MHz.

These experiments mimicked the conditions of a device implanted subcutaneously in mice and evaluate the feasibility of the FUS thermal-actuation. For this reason the devices were sandwiched between a layer of mouse skin on the top and a piece of chicken tissue in the bottom. The devices were placed with the apertures facing down (towards the chicken tissue), so the dextran release took place in the same direction that the ultrasound. The geometric focus of the transducer was manually aligned with the implant using the ultrasound-echo signal obtained from the diagnostic transducer. During the localized heating of the implant, the FUS transducer was operated in a quasi- continuous wave (CW) mode (<1 kW/cm^2) and the diagnostic transducer in pulse-echo mode at a PRF of 100 Hz to perform noninvasive thermometry (pulser-receiver system, Model 5052PR, Panametrics). Local temperature change was inferred from the thermally induced change in sound speed and consequently in a time-shift of the echo signal. The noninvasively obtained local temperature was used as a feedback to modulate the FUS amplitude for maintaining the temperature of the implant at 45 ± 0.5 ºC.

For the ex-vivo experiments AlexaFluor-dextran 680 (10 kDa) was used as a drug

substitute to be released from the NipAAm capsules. AlexaFluor 680 was chosen because it has an excitation wavelength of 679 nm and an emission wavelength of 702 nm; making it very suitable to obtain good quality fluorescent images as at those wavelengths the auto-fluorescent background from tissue is minimal.

Animal experiments

One sterile device was implanted subcutaneously in the dorsum of 8 male athymic nude mice (NCr nude, Taconic) under isoflurane anesthesia. The incision (≈2cm) was closed using surgical staples and liquid pockets were created around the device injecting PBS saline buffer. During FUS actuation and in-vivo imaging, the mice were anaesthetized using isoflurane, so the animals did not have to be restrained. All animals were at least for a week before surgery on alfalfa-free diet (Teklad Global Diet, 2914) to minimize autofluorescent signals from the digestive track in the far-red range during in vivo imaging. All animal procedures were performed in accordance with approved Institutional Animal Care and Use Committee protocols.

For histological studies, the PDMS capsules were implanted for 2 and 4 weeks for the H&E and Trichrome analysis without FUS treatment. Tissue samples were excised at the time of device explant and stored in 10% buffered formalin for 24 hours prior to analysis. One cross perpendicular sections passing through the center of the tissue sample was taken and embedded in paraffin. Histologic slides were prepared and stained with hematoxylin and eosin (H&E), trichrome or TUNEL assay. Using the same segmentation thresholds for FUS-treated and control images, percentage of apoptotic cells was determined as the ratio of number of pixels representing apoptotic cells over total cell-related pixels.

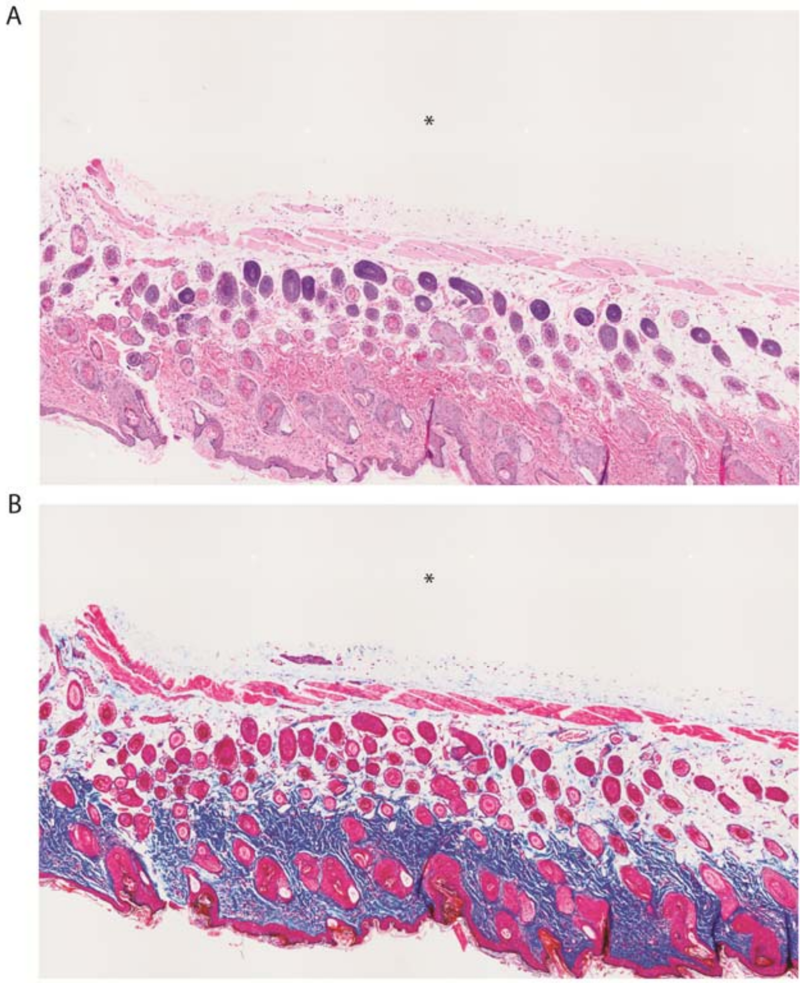

Figure S3. Histology of mouse skin after implantation of iTAG and FUS actuation. Excised skin from the mouse was stained with hematoxylin and eosin *(A)* as well as trichrome *(B)*. Histological analysis revealed minimal presence of giant body cells indicating the lack of chronic inflammation. A thin fibrous capsule (<1mm) was also present indicating normal wound healing. The asterisk (*) indicates the location of the device *in vivo*
